# Supplementary figures and images for: The cells are all-right: Regulation of the Lefty genes by separate enhancers in mouse embryonic stem cells
Source: PLoS Genet. 2024 Dec 13;20(12):e1011513. doi: 10.1371/journal.pgen.1011513 (PMC11676945; doi:10.1371/journal.pgen.1011513)

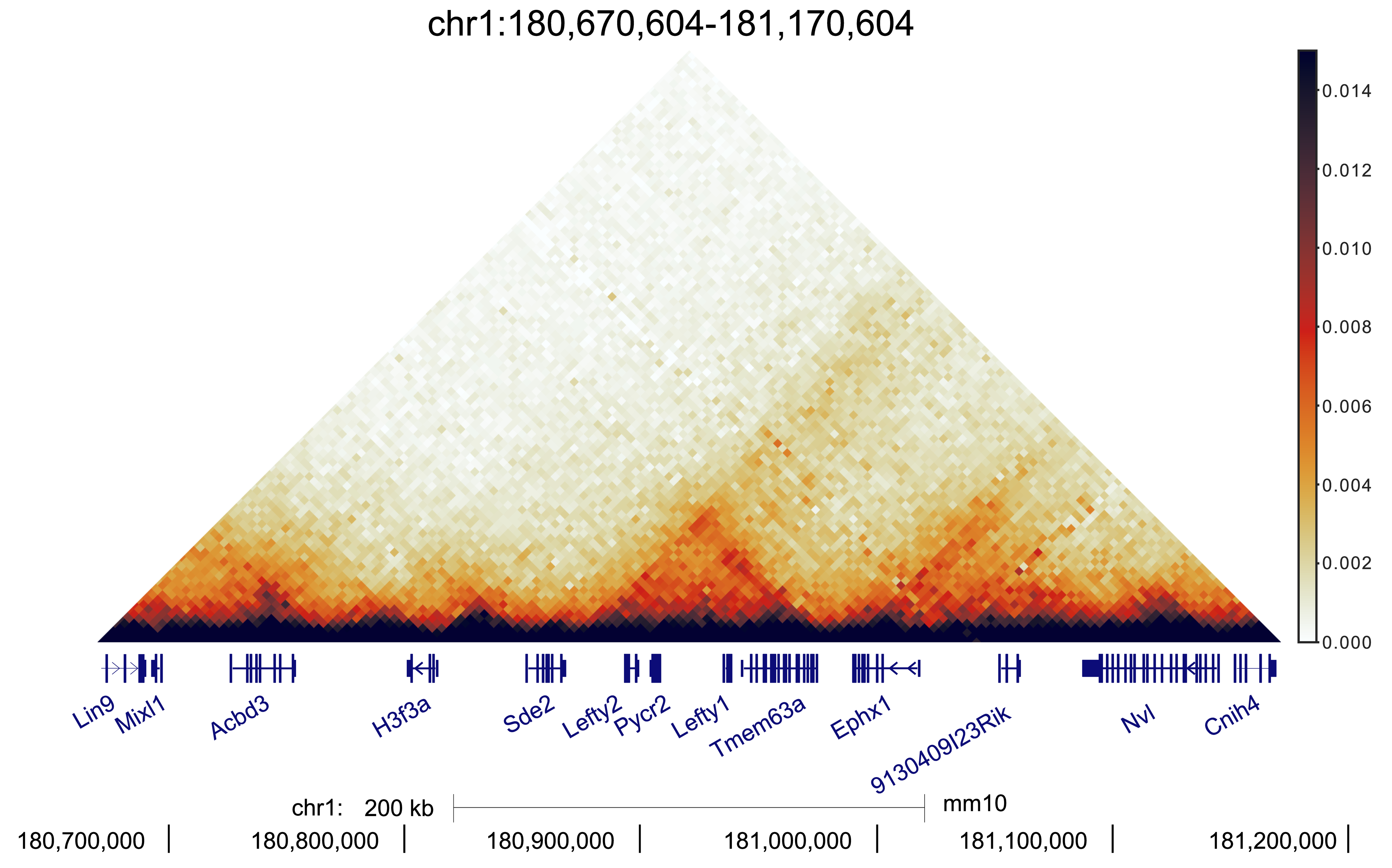

Supplement: S1 Fig — Genes are represented along the bottom of the heatmap. The Lefty genes reside within a shared interacting region. (TIF) [file pgen.1011513.s001.tif]

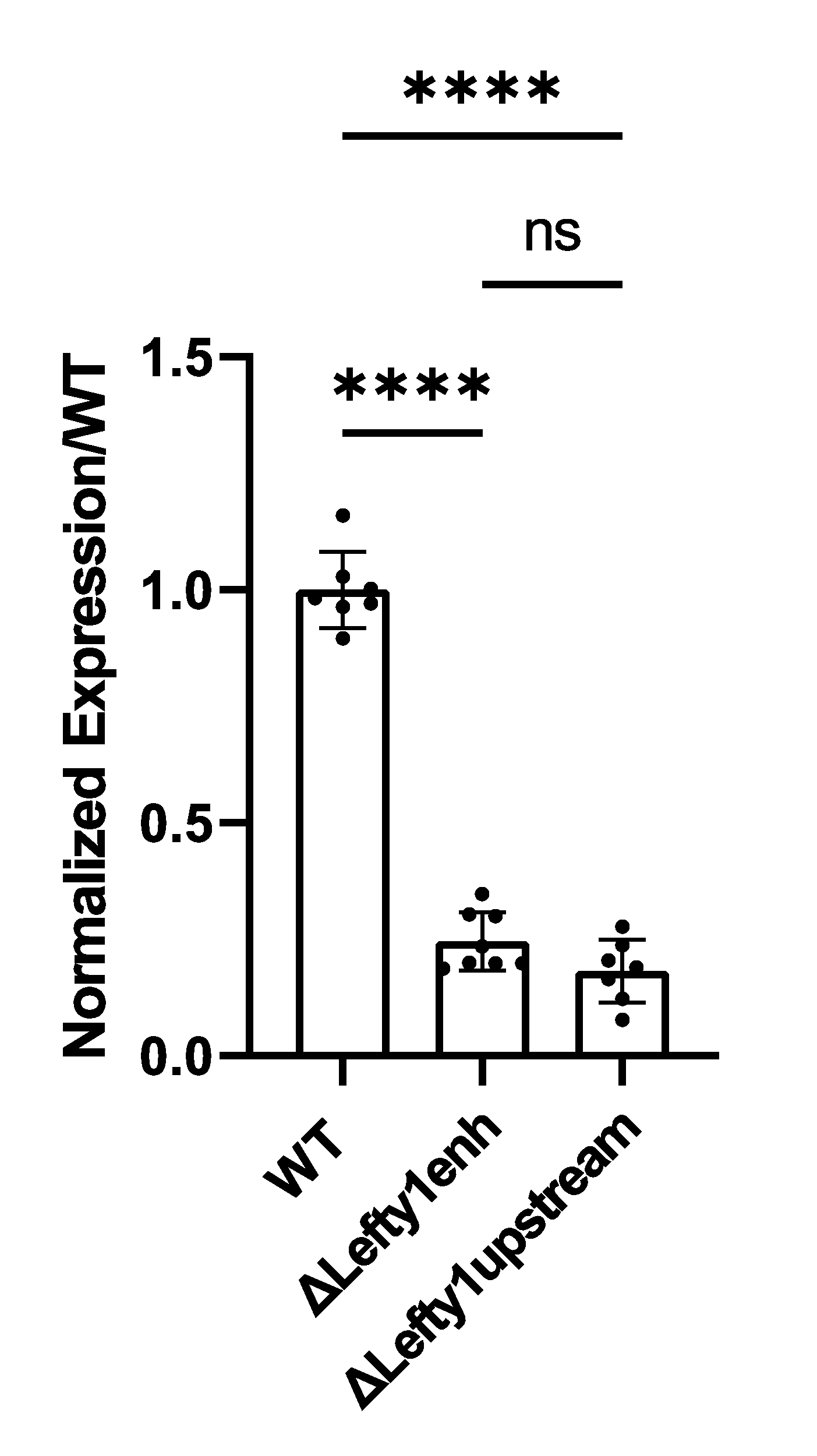

Supplement: S2 Fig — No Significant difference in expression is observed in Lefty1 expression between the two deletions, both are significantly reduced in expression as compared to wild-type cells. Error bars represent the SD, one way ANOVA significant differences are indicated. (*) P < 0.05, (**) P < 0.01, (***) P < 0.001, (****) P < 0.0001, (ns) not significant. (TIF) [file pgen.1011513.s002.tif]

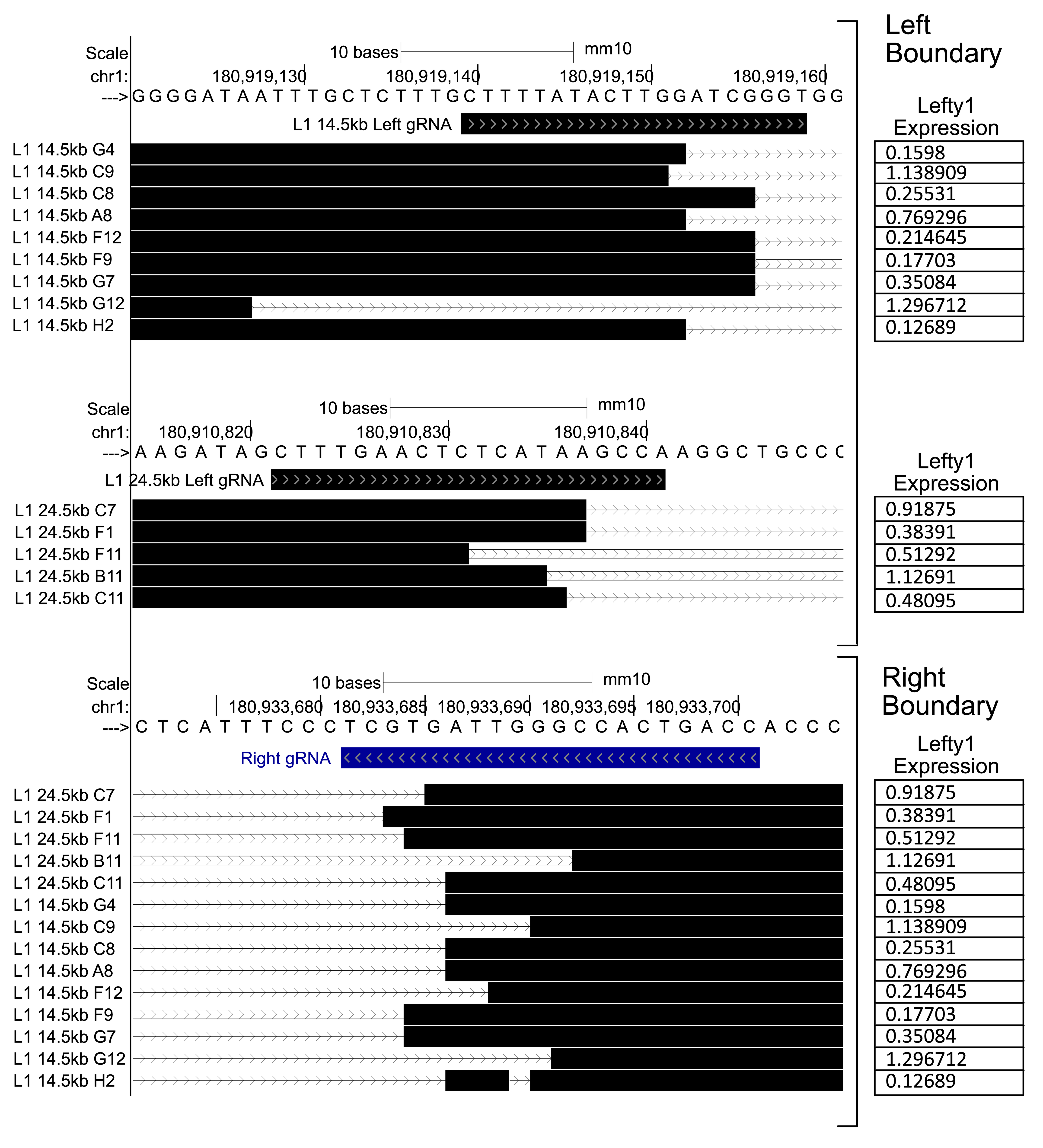

Supplement: S3 Fig — The precise breakpoints of L1 14.5kb and L1 24.5kb deletions varied by <32bp, differences in deletions boundaries do not seem to correlate with the clonal variability in Lefty expression which was observed. Values of Lefty1 expression by clone are shown in the table on the right, the values are shown as a proportion of wild-type expression. Clone names are shown beside the left or right boundary of the sequenced deletion, dark bars show the remaining aligned genomic sequence whereas the thin lines represent the removed region. (TIF) [file pgen.1011513.s003.tif]

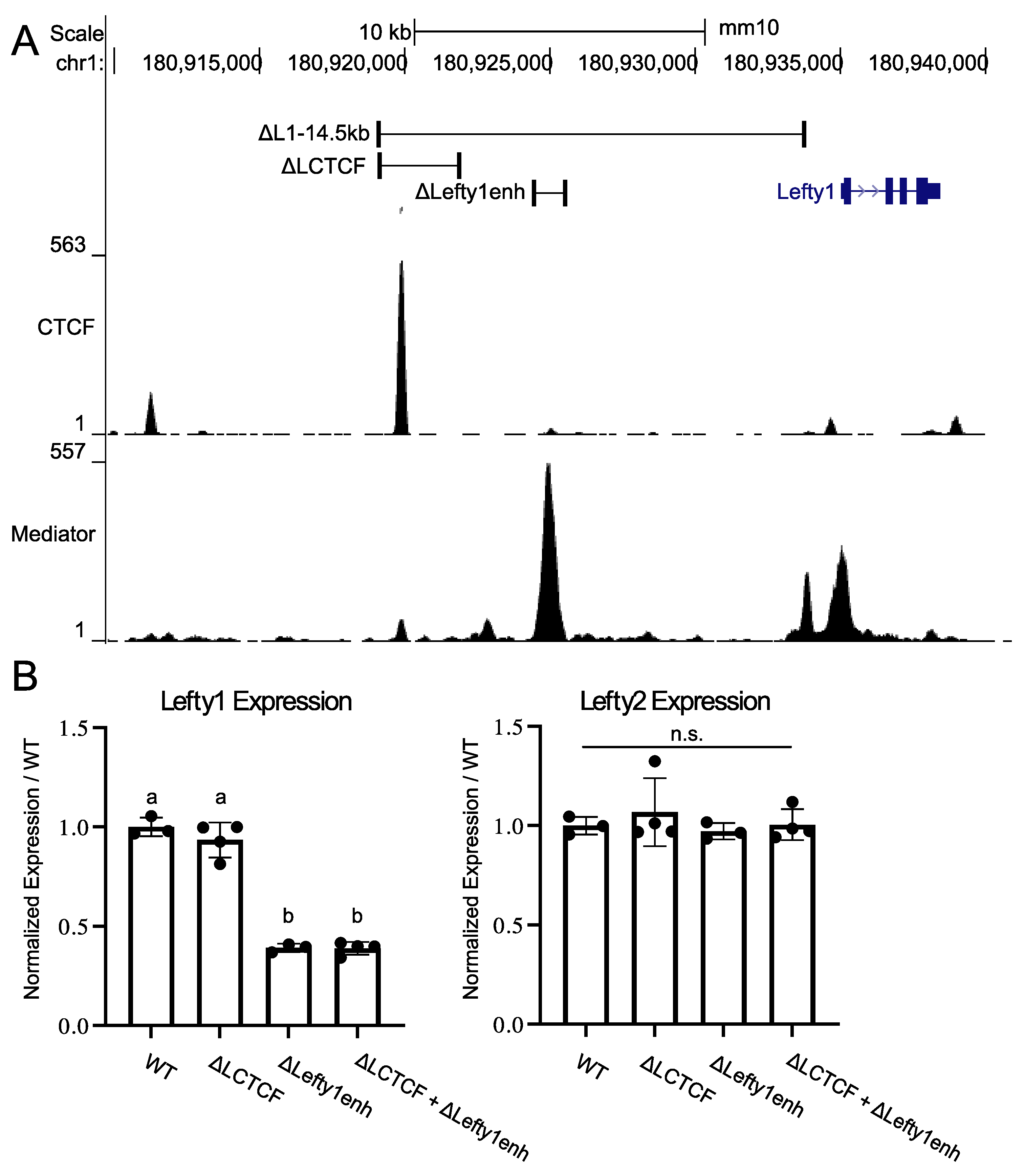

Supplement: S4 Fig — A) UCSC browser view (mm10) of the region upstream of Lefty1 highlighting the previous deletion of the entire upstream region which showed variable Lefty1 expression (ΔL1-14.5kb) compared to the smaller deletion of the CTCF bound region (ΔLCTCF) and the Lefty1 enhancer (ΔLefty1enh) deletion. ChIP-seq data for CTCF binding and Mediator (Med1) shown to highlight regulatory versus architectural protein binding locations. B) Expression of Lefty1 and Lefty2 in CTCF and Lefty1 enhancer deleted cell lines is shown fold change compared to WT expression. Error bars represent the SD, significantly different groups are highlighted as identified by one-way ANOVA. (TIF) [file pgen.1011513.s004.tif]

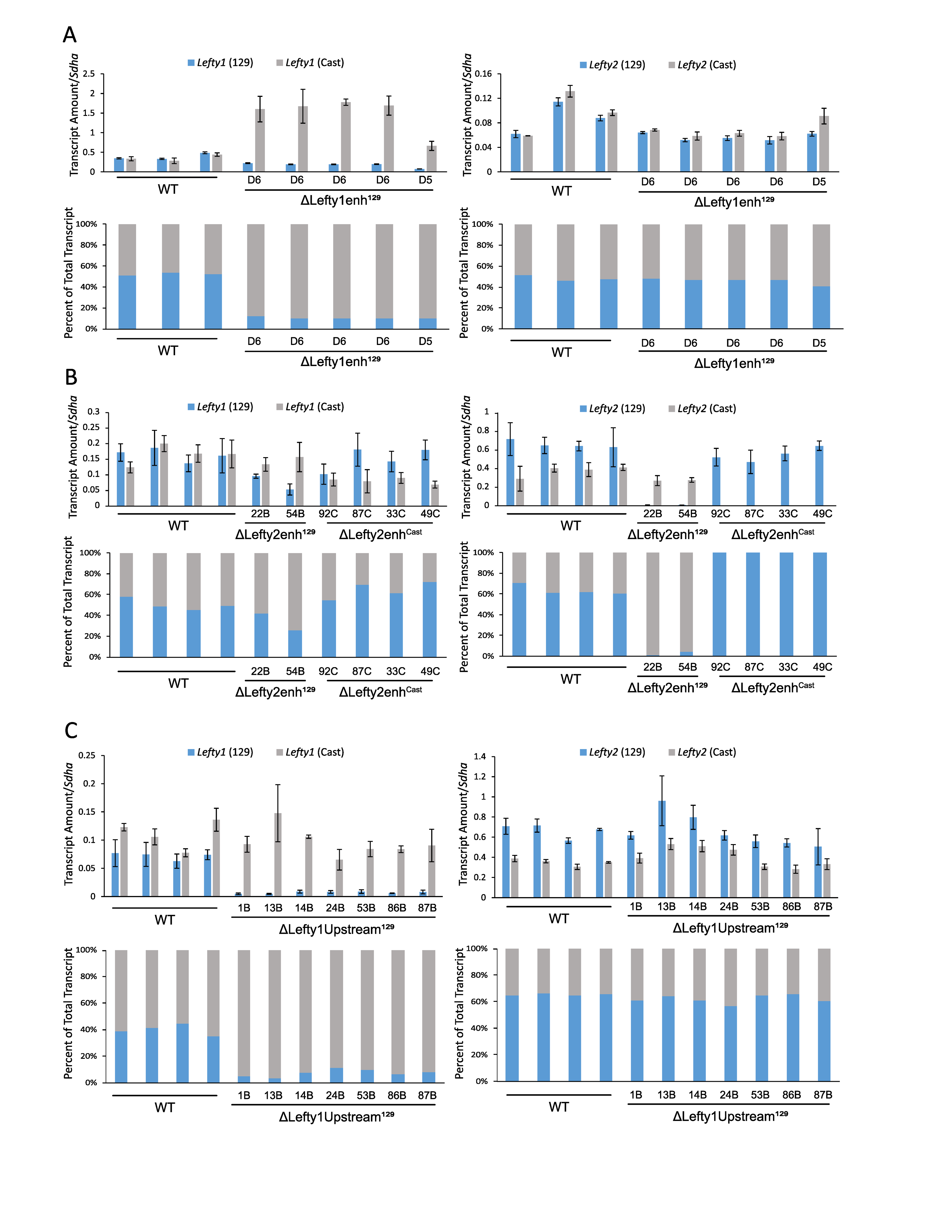

Supplement: S5 Fig — Comparison of Lefty1 (Left) and Lefty2 (right) expression across individual enhancer deleted clones. Expression is shown as transcript amount normalized to Sdha as calculated using the standard curve method or as allele-specific ratio of the proportion of expression of either allele over the total expression. Error bars represent the standard deviation of technical replicates. A) Deletion of the Lefty1 enhancer on the 129 allele appears to cause a compensatory increase in Lefty1 expression from the Castaneus allele. B) Deletion of the Lefty2 enhancer leads to an allele-specific loss of Lefty2 expression with no sign of compensation by the intact allele. C) A larger deletion targeting the Lefty1 upstream region encompassing the enhancer leads to a loss of Lefty1 expression with no evidence of compensation by the intact allele. (TIF) [file pgen.1011513.s005.tif]
