# Supplementary material for: The cells are all-right: Regulation of the Lefty genes by separate enhancers in mouse embryonic stem cells
Source: PLoS Genet. 2024 Dec 13;20(12):e1011513. doi: 10.1371/journal.pgen.1011513 (PMC11676945; doi:10.1371/journal.pgen.1011513)
Supplement: Attachment — Submitted filename: Taylloe_Sept_18_2024 Revision Plan and Response Combined.docx [file pgen.1011513.s010.docx]

**Manuscript number:** RC-2024-02571

**Corresponding author(s):** Jennifer, Mitchell

## 1. General Statements

We greatly appreciate the feedback and suggestions from the reviewers. We believe that all reviewers had a strong understanding of the article and gave constructive feedback which only serves to improve the final product. Our goal was to investigate the relationship between chromatin architecture and cis-regulatory crosstalk in the *Lefty* locus and the suggestions by reviewers will be incorporated to clarify that goal.

## 2. Description of the planned revisions

- Reviewer 2 pointed out that a more targeted deletion of the CTCF bound region would clarify to which extent CTCF or distance was involved in the insulation we observed for the *Lefty* genes. This is an excellent point and to address this we will create a new targeted deletion which removes the most prominent CTCF bound region located upstream of the *Lefty1* enhancer, both on its own and alongside deletion of the *Lefty1* enhancer itself. This is a compound deletion which will remove these two regions separately while leaving the rest of the locus intact to independently test the effects of CTCF vs genomic distance on *Lefty* crosstalk as suggested by reviewer #2. We expect these deletions to take 4-6 weeks to obtain, genotype and evaluate the effect on expression in multiple clones. The findings from this approach should clearly reveal if CTCF, distance or an effect of both is involved in insulating the *Lefty* genes.

## 3. Description of the revisions that have already been incorporated in the transferred manuscript

- We have more clearly defined our terminology for “architectural insulation” to be defined as a clear boundary or absence of interaction between the *Lefty* genes as is seen at TAD boundaries. We do detect an interaction between the two *Lefty* enhancers, but as pointed out by reviewer 2 this does not mean they are not insulated to some extent from one another by chromatin conformation. We have changed instances where we previously stated that we do not observe architectural insulation to instead say that we observe a lack of a clear boundary between the genes. Suggestion by reviewer #2
- We have revised our statement "Interestingly, we note that the remaining *Lefty1* expression in the *Lefty1* enhancer deleted cell line is similar to the decrease seen when the *Lefty2* enhancer was deleted, highlighting that although the *Lefty2* enhancer may exert some regulatory control over the *Lefty1* gene, it does not act in a functionally redundant or synergistic manner with the *Lefty1* enhancer." As pointed out by reviewer 2 this may be coincidental and we have revised our statement as follows; “We note that the remaining *Lefty1* expression in the *Lefty1* enhancer deleted cell line is similar to the decrease seen when the *Lefty2* enhancer was deleted. Although this may be coincidental, it suggests *Lefty1* is regulated by both the *Lefty2* and *Lefty1* enhancers in a roughly additive manner.” Suggestion by reviewer #2
- We have added clarifications to the methods as to how the gene expression normalization approach was carried out and to clearly delineate what the values of the Hi-TrAC plots mean. For gene expression normalization, we have explained that the values shown represent the allelic ratio of expression from a deleted clone compared to the ratio of expression in wild-type cells. We have included a figure (supplemental figure 4) which shows the non-normalized values, both the transcript quantity detected by qPCR and the calculated allelic ratios for individual enhancer knockout clones. We have expanded upon the figure legend for Figure 2 to explain what data is being represented in the virtual 4C plots. We have expanded upon the Hi-TrAC section of the methods to list all of the datasets included in the figure and which parts of the figure were generated by cLoops2. Suggestions by reviewer #2
- We have generated a supplemental figure (supplemental figure 4) with the expression of the *Lefty* genes from either allele for clones containing deletions of either *Lefty* enhancer to compare gene dosage compensation from either allele when an enhancer is removed. Suggestion by reviewer #2
- We have added to the discussion that the methods for detecting chromatin contacts that we used only measure pairwise interactions and cannot detect multiway interactions or temporal chromatin dynamics Suggestion by reviewer #3

## 4. Description of analyses that authors prefer not to carry out

- The focus of this investigation was the cis-regulatory cross talk between the *Lefty* genes. Although interesting we believe it is not within the scope of the article to study the upstream trans-regulators of the *Lefty* enhancers using a transcription factor degron or deletion approach. In addition, there are confounding factors in deletion/depletion approaches in ESCs as many of the factors bound at the two enhancers are themselves factors that maintain cell identity and their depletion would cause cell differentiation likely to obscure the identification of the upstream regulators. (Suggestion by reviewer #3)
- Reviewer 3 suggested we investigate a potential hub-like formation of this locus in the floor plate or lateral plate mesoderm. Although interesting this is a highly technically challenging experiment. The developing mouse embryo at the timepoint where these tissues are present is comprised of approximately 20,000 cells, these targeted tissues contain only hundreds to thousands of cells and would be difficult to isolate without fluorescent markers of the cell types. In addition, since the focus of our study was the cis-regulatory cross talk between the *Lefty* genes in pluripotent ESCs and we have not investigated a hub-like formation in this context nor indicated that the *Lefty* locus appears to have a hub structure we believe this experiment is outside of the scope of our study.

# Full Review Section and Response to Reviews

------------------------------------------------------------------------------

**Reviewer #1 (Evidence, reproducibility and clarity (Required)):**

Taylor et al. present an in-depth analysis of regulatory DNAs in the lefty1/2 locus of the mouse, with a particular emphasis on the regulation of these genes in mESCs.

The study is nicely executed and reminds us that linked paralogs are not necessarily co-regulated, but can exhibit distinct profiles of gene activity even when closely associated.

**Reviewer #1 (Significance (Required)):**

The authors identify two new enhancers, Enh2 and Enh1, which are located upstream of lefty2 and lefty1, respectively. Both enhancers mediate expression in mESCs and Hi-C contact maps suggest that they interact with one another. Nonetheless, there is surprisingly little cross-regulation of the two genes despite their relatively close proximity within a common TAD.

The authors examine the consequences of deleting a variety of sequences in the 44 kb interval separating the two genes. A deletion that places the Enh2 enhancer in close proximity with the lefty1 promoter leads to a significant increase in its expression. These and other observations suggest that the Enh2 enhancer and lefty1 promoter are compatible, but in the context of the native locus there is only limited interactions between the two. It would therefore appear that physical separation of lefty2 and lefty1 is sufficient for independent regulation of the two genes.

**RESPONSE:** We thank reviewer #1 for their kind comments and accurate description of the studies findings.

**Reviewer #2 (Evidence, reproducibility and clarity (Required)):**

Summary:

This manuscript by Taylor et al. investigates the differential regulation of the two paralogous Lefty genes in mESCs, that arose through a gene duplication event. They identify new enhancers, the Lefty1 enhancer and the Lefty2 enhancer, that explicitly regulate their target promoters in the endogenous set-up of the locus. Through clever CRISPR/Cas9 deletions in the Lefty locus, the authors show that the Lefty2 enhancer can generally also activate the Lefty1 promoter, however the intervening sequence serves an insulator to prevent aberrant activation in the endogenous context. The study then further identifies features of the intervening sequence that confer this insulation, specifically looking at architectural insulation, CTCF binding sites and the genomic distance at which the enhancer and promoter are placed.

**RESPONSE:** We thank reviewer #2 for their accurate description of the manuscript and kind comments about the approach

Major comments:

1. The term "architectural insulation" should be defined more clearly. Based on the statements of the authors I understand the term as an absence of contact probabilities between enhancer and promoter in Hi-TrAC or micro-C maps that are higher than expected from a simple power-law decay with genomic distance. In the study, they find higher than expected contact frequencies between the enhancers and the promoters (i. e. a "contact") and they find the Lefty enhancers and Lefty genes to be located within the same TAD, concluding that there is no architectural insulation between the enhancers and promoters. However, such contacts, as well as the fact that they are located in the same TAD, do not exclude the possibility of insulation through e. g. formation of CTCF loops not anchored directly at the enhancer/promoter (such loops can be seen in the provided contact maps). This could ultimately affect contact frequencies between the enhancer and the promoter and also transcriptional levels (see deletion contexts). Insulation through the architecture of the 3D genome is therefore still possible through a modulation of their contact frequencies.

**RESPONSE:**

- This is a good point by the reviewer and we apologise for the lack of clarity. We have adjusted the text accordingly to better define “architectural insulation” to refer to the presence of a clear boundary, similar to those observed at the borders of TADs, between the two genes. The text now reads, “We next asked whether the specificity in regulation by the *Lefty* enhancers was due to a strong architectural insulation of the two genes, segregating them into separate TADs.”
- We agree that we cannot exclude the possibility that contact frequencies within the locus are affected by CTCF bound regions, only that we do observe peaks in contact between the two *Lefty* enhancers and genes as displayed in Figure 2. To better reflect these nuances, we have added the following to the discussion; “In addition, we show that these two genes and their cognate ESC enhancers interact in conformation capture assays that show no evidence of a strong architectural boundary between the two genes. Despite this apparent lack of architectural insulation, we find that the 44kb of chromatin separating these two genes does transcriptionally insulate the *Lefty2* enhancer from the *Lefty1* gene, however, we cannot rule out that intervening elements may be acting to attenuate a stronger architectural interaction between these genes.”

2. In Figure 5, the authors perform a series of deletions of increasingly larger portions of the intervening sequence between the Lefty2enh and the Lefty1 gene. The main conclusions from these experiments are that the CTCF-bound sites as well as the genomic distance contribute to the insulation between Lefty2enh and Lefty1 promoter. However, the set-up of these experiments cannot distinguish the effects of CTCF site insulation and the effect of genomic distance, as both factors are changed in the same experiment when deleting a large portion of the locus that contains CTCF sites. To clearly identify the contribution of CTCF bound sites to the insulation, the authors should consider deleting only the CTCF sites, keeping the surrounding sequence constant. This experiment is highly feasible, as it uses the same techniques needed for the experiments described in the manuscript (CRISPR/Cas9 deletions and quantification of transcription by allele-specific qPCR).

**RESPONSE:**

- We agree that more focused deletions of CTCF bound regions would clarify this point. In the L1-31kb deletion where we observed *Lefty1* expression levels return to normal in the absence of the *Lefty1* enhancer, indicating control by the *Lefty2* enhancer only two CTCF bound regions were removed. As suggested by the reviewer we propose, to create a new deletion removing the larger CTCF peak alone and in combination with the *Lefty1* enhancer, but in subsequent deletions, to better separate the effects of distance from CTCF deletion. If the CTCF bound region is important we expect to see a change in expression similar to what was observed in the L1-14.5kb deletion. If we observe a change, we will subsequently remove the less prominent CTCF bound region in these cells to observe any potential compound effect.

3. The claim that the genomic distance itself is a determining factor should be revisited. In comparable studies cited also by the authors, such as Rinzema et al. 2022, PMID: 35710842 (but see also Zuin et al. 2022, PMID: 35418676), the experimental set-up is conceptually different: There, the enhancer is moved at varying distances to the promoter, while retaining the intervening sequence (deletion and reinsertion of the enhancer within the locus). With this experimental approach the effect of genomic distance can be unambiguously studied. However, in this study, the intervening sequence is deleted, thereby changing not only the distance but also the sequence identity. Since this could contribute to the insulation (such is hinted at for the parts that contain CTCF sites), it is difficult to determine the contribution of the genomic distance with this experimental set-up. Deleting pronounced features such as the CTCF bound sites while keeping the distance constant, could help to distinguish between the effect of sequence features vs. the genomic distance and would make the conclusions drawn in this manuscript stronger.

**RESPONSE:**

- We acknowledge that the reductionist approach we have taken, removing intervening sequence, varies conceptually from approaches which move an enhancer to different distances from the promoter by insertion. However, we argue that both approaches suffer from the concern of changing the sequence identity for regions between the two features. We do agree that more focused CTCF bound region deletions would help to clarify this point and we will conduct these as detailed above.

4. OPTIONAL: An interesting observation is the high levels of variability in expression for the deltaL1-14.5kb and deltaL1-24.5kb deletions. It stands to reason that this is a direct effect of loss of insulation by CTCF, as these are the deletions that remove the CTCF bound sites. How does the variability in expression for these clones compare to a direct knock-out of the CTCF sites only?

**RESPONSE:**

- This will be compared in the new deletion that will be created, as described above. We agree that comparing the larger deletion to the more focused CTCF site deletion will clarify the mechanism.

Minor comments:

1. The following statement should be revised: "Interestingly, we note that the remaining Lefty1 expression in the Lefty1 enhancer deleted cell line is similar to the decrease seen when the Lefty2 enhancer was deleted, highlighting that although the Lefty2 enhancer may exert some regulatory control over the Lefty1 gene, it does not act in a functionally redundant or synergistic manner with the Lefty1 enhancer." The fact that the expression levels of Lefty1 in the Lefty1enh deleted line and the Lefty2enh deleted line add up to 100%, does not mean that the enhancers do not act synergistically. In fact, in both deletion backgrounds, a number of other factors are changed upon deletion and could further contribute to the observed expression levels, such as modulation in the Lefty1enh strength upon deletion of Lefty2enh, a modulation in expression levels of intervening genes, etc. That the observed expression levels in deletion backgrounds together yield WT expression levels might therefore be just a coincidence.

**RESPONSE:**

- We have revised this statement to reflect these nuances, the text now reads; “We note that the remaining *Lefty1* expression in the *Lefty1* enhancer deleted cell line is similar to the decrease seen when the *Lefty2* enhancer was deleted. Although this may be coincidental, it suggests *Lefty1* is regulated by both the *Lefty2* and *Lefty1* enhancers in a roughly additive manner.”

2. The methods section as well as the figure captions could be more detailed, e.g. the normalization strategy for the expression levels is difficult to understand. Furthermore, the description of the analysis of the Hi-TrAC data with an explanation of the values plotted in Fig. 2 could be more extensive.

**RESPONSE:**

- Thank you for pointing out that this was not clear, we have clarified these points further in the text and in response to the optional point below.

3. OPTIONAL: It would be interesting to also show the expression levels of the non-edited allele to describe dosage compensation strategies in trans at loci that contain duplicated genes. Specifically, in the absence of one of the enhancers and a down-regulation of the gene it regulates, how is the expression of said gene on the WT allele affected?

**RESPONSE:**

- We have generated supplemental figure S4 with this information which we hope also clarifies the initial steps in the normalization strategy.
- In our deletion approaches we use the intact allele as an internal control as overall gene expression levels can be variable between experiments whereas the allelic-ratio of expression is more consistent (Zhou *et al.* 2014, Moorthy *et al.* 2017, Taylor *et al.* 2022). This allows us to evaluate finer scale changes in gene expression after enhancer deletion.
- After quantifying each allele in both WT and deleted clonal isolates, with technical replicates in the qPCR (error bars in the top graphs for S4A/B/C), we next express the data for each allele as a percentage of the total transcript amount (bottom graphs for S4A/B/C) obtained by summing the amounts from each allele. This is supported by our qPCR approach which uses genomic DNA to create standard curves for absolute quantification of each allele in RNA samples. The graphs in the main manuscript use the individual clonal isolates as biological replicates. To facilitate the comparison between experiments we normalize the percent expression from the deleted allele to the percent expression of the matched WT allele.
- The graphs in figure S4 show that we don’t observe any evidence of compensation by the non-deleted allele except in clone D6 for the Lefty1enh deletion (figure S4A). Note for that clone we had multiple samples that were independently grown and analysed all showing this higher expression of the non-deleted allele (Cast). However, when the allelic ratio was calculated any variation between clones for either WT or the deletion was lost (S4A bottom graphs).

REFEREE CROSS-COMMENTS

The comments of the other reviewers are largely in line with my own comments. The additional points raised by reviewer 3, in particular the idea to study the locus in differentiated cells to point to a function of the 3D configuration, seem reasonable.

**RESPONSE:** Please see our response to the comment below.

**Reviewer #2 (Significance (Required)):**

The presented data in this manuscript is scientifically sound and most of conclusions drawn from it are well supported. However, the concluding statements on features that contribute to the insulation effect between the Lefty1 gene and the Lefty2 enhancer should be clarified as indicated in the comments. A clear distinction of the insulating effects resulting from the genomic distance vs. from the sequence identity would strengthen the message of this manuscript.

The study is timely, as it contributes to the ongoing conversations on factors that define enhancer-promoter selectivity, be it their inherent compatibility for each other or influences of the surrounding sequence, such as the 3D genome architecture of the locus. The observations made on compatibility between the Lefty enhancers and their target genes as well as on other genes in this locus can serve as benchmarks in future studies that investigate enhancer-promoter compatibility. Furthermore, the deletion experiments are an important independent data set on how genomic distance between enhancer-promoter pairs can affect transcriptional regulation; an area of transcriptional regulation that is not yet well understood and where few studies have been published (Symmons et al. 2016, PMID: 27867070, Rinzema et al. 2022, PMID: 35710842, Zuin et al. 2022, PMID: 35418676, Thomas et al. 2023, doi: https://doi.org/10.1101/2023.12.06.570399). I expect that researchers studying fundamental questions in the field of gene regulation will be interested in this study.

My own background is in the field of gene regulation and chromatin architecture. I can therefore only assume that this study will also be of interest to researchers in evolutionary biology, since the locus that was chosen here, presents an interesting case from an evolutionary perspective: It shows how transcriptional regulation is adapted to account for the higher dosage in duplication events. However, besides choosing the locus, the authors of the manuscript do not elaborate on what these results mean for gene dosage compensation.

**RESPONSE:**

- We would like to thank reviewer #2 for their comments and critiques of the article. We believe that their recommendations serve to better clarify and strengthen the data we have presented, and we greatly appreciate their insight.

Reviewer #3 (Evidence, reproducibility and clarity (Required)):

Summary

Taylor et al present a study aimed at understanding regulation of duplicated Lefty1 and Lefty2 genes in embryonic stem cells. The regulation of these genes and the architectural organization of the locus encoding them are interesting because although closely related and located within the same topologically associated domain (TAD) , they appear to be independently regulated in some tissues. Both Lefty 1 and Lefty2 are expressed in ESC and are involved in pluripotency maintenance, however, in terms of driving right/left patterning during embryonic development, Lefty1 is primarily expressed in the prospective floor plate and Lefty2 is expressed in the lateral plate mesoderm. These authors use CRISPR-Cas9 allele-specific genome editing of an F1 mouse ESC line (Mus musculus x Mus casteneus) to localize enhancer elements for each gene and then test their specificity in ESC. The enhancer search, carried out using CRISPR-Cas9 mediated deletion, identified elements overlapping motifs for core ESC pluripotency transcription factors, upstream of each gene and overlapping a previously identified asymmetric specific enhancer (ASE) for Lefty1 and near the previously identified neural plate enhancer (NPE) and lateral plate mesoderm-specific enhancer (LPE) for Lefty2. The authors highly specific approach showed almost total but incomplete regulation of Lefty1 by its putative enhancer and substantial specificity of the putative Lefty2 enhancer for Lefty2. Insulation, or long distance interactions, directed by CTCF sites between the genes was not a factor in their distinct regulation according to structural analysis using Hi-TrAC but further analysis using virtual HiC showed that the enhancers interact with each other in the nucleus and form various other hub-like interactions with the genes. Finally, CRISPR-Cas9 deletions of various lengths between the genes supplies support for the idea that separation of the genes/enhancers from each may plays a role in their regulation in ESC.

**RESPONSE:** We would like to thank reviewer #3 for their accurate description of the findings from the article

Comments

1. It would strengthen the arguments presented for enhancer function of the identified sequences if ESC with reduced transcription factor expression (deletion, auxin-depletion) were examined.

**RESPONSE:**

- It is an interesting idea to use a degron approach to identify upstream regulators of the *Lefty* genes; however, the focus of this investigation was the cis-regulatory cross talk between the *Lefty* genes. Although interesting we believe it is not within the scope of the article to study the upstream trans-regulators of the *Lefty* enhancers using a transcription factor degron or deletion approach. In addition, there are confounding factors in deletion/depletion approaches in ESCs as many of the factors bound at the two enhancers are themselves factors that maintain cell identity and their depletion would cause cell differentiation likely to obscure the identification of the upstream regulators.

2. The hub like interactions of the Lefty locus in ECS is interesting and one of the approaches that allows more than pairwise interactions to be detected would be helpful to map out which interactions are simultaneous. This issue should at least be discussed.

**RESPONSE:**

- This is a very interesting point and one that we had not considered. The ligation-based interaction data that we processed identified only pairwise interactions between any two regions. Other ligation-based methods exist which allow for the identification of multiway chromatin interactions, Tri-C for example, however the publicly available data for these methods does not encompass the *Lefty* locus and we feel that conducting these experiments fall outside the scope of a revision since our focus was on investigating the *cis*-regulatory cross talk between these 2 genes. Understanding the dynamics of chromatin architecture has been examined using microscopy-based techniques, however, the *Lefty* locus is too small in size to be able to resolve changes in distance using fluorescent probes. Although we don’t know if these interactions happen simultaneously, the transcription of these genes may occur independent of enhancer proximity as in (Alexander et al., 2019; DOI: 10.7554/eLife.41769). Detection of a multiway interaction may not correlate with transcriptional bursts of the *Lefty* genes.
- As the reviewer has suggested we have now discussed the potential for multiway interactions in the article discussion.

3. the hub-like formation of this locus be examined in differentiated cells like floor plate or lateral plate mesoderm? This could possibly invoke a function for the 3D conformation of the locus.

**RESPONSE:**

- Although interesting this is a highly technically challenging experiment. The developing mouse embryo at the timepoint where these tissues are present is comprised of approximately 20,000 cells, these targeted tissues contain only hundreds to thousands of cells and would be difficult to isolate without fluorescent markers of the cell types. In addition, since the focus of our study was the cis-regulatory cross talk between the *Lefty* genes in pluripotent ESCs and we have not investigated a hub-like formation in this context nor indicated that the *Lefty* locus appears to have a hub structure anywhere in our manuscript we believe this experiment is outside of the scope of our study.

**Reviewer #3 (Significance (Required)):**

This work is significant because it provides a much more molecular dissection of the Lefty enhancers that Lac-Z expression studies. It also attempts to discern what elements of chromatin folding affect the specificity of Lefty gene enhancer regulation. They uncover a role for distance between the enhancer/gene pairs in conferring specificity.

**RESPONSE:** We would like to thank reviewer #3 for their feedback and comments about the article, we believe their observations serve to improve the article.
